# Multifaceted effect of chlorpromazine in cancer: implications for cancer treatment

**DOI:** 10.18632/oncotarget.28010

**Published:** 2021-07-06

**Authors:** Pareesa Kamgar-Dayhoff, Tinatin I. Brelidze

**Affiliations:** ^1^Department of Pharmacology and Physiology, Georgetown University Medical Center, Washington, D.C., USA

**Keywords:** chlorpromazine, repurposing

## Abstract

Since its discovery in 1951, chlorpromazine (CPZ) has been one of the most widely used antipsychotic medications for treating schizophrenia and other psychiatric disorders. In addition to its antipsychotic effect, many studies in the last several decades have found that CPZ has a potent antitumorigenic effect. These studies have shown that CPZ affects a number of molecular oncogenic targets through multiple pathways, including the regulation of cell cycle, cancer growth and metastasis, chemo-resistance and stemness of cancer cells. Here we review studies on molecular mechanisms of CPZ’s action on key proteins involved in cancer, including p53, YAP, Ras protein, ion channels, and MAPKs. We discuss common and overlapping signaling pathways of CPZ’s action, its cancer-type specificity, antitumorigenic effects of CPZ reported in animal models and population studies on the rate of cancer in psychiatric patients. We also discuss the potential benefits and limitations of repurposing CPZ for cancer treatment.

## INTRODUCTION

Chlorpromazine (CPZ), a member of the thiazine-class of heterocyclic compounds known as phenothiazines, appeared in the repertoire of psychotherapeutic drugs in 1952, revolutionizing the treatment of psychiatric disorders [1, 2]. Before the discovery of CPZ, the treatments for psychiatric disorders were limited to either invasive therapies, such as insulin comas and electroconvulsive therapy, or non-specific and addictive pharmacological agents such as opium, morphine and cocaine [3, 4]. Although CPZ is a first-generation antipsychotic medication, it is still widely used and several recent studies found that the latest (second) generation antipsychotic drugs do not provide substantial advantages for patient treatment [5, 6].

Not long after CPZ entered clinical use as an antipsychotic, it was proposed that CPZ also possessed anticancer activity. Epidemiological studies conducted in Denmark from 1957 to 1980 suggested that psychiatric patients prescribed CPZ had decreased risk of developing cancer [7]. In 1972, a case report indicated a significant inhibition of tumor growth in a patient with squamous-cell carcinoma of the larynx after directly injecting CPZ into the tumor [8]. Anticancer effect of CPZ was also observed in animal studies. It was reported that CPZ and other structurally similar phenothiazines inhibited sarcoma tumor growth in mice [9–11]. Importantly, the antitumor potency of the antipsychotic drugs did not correlate with their antipsychotic activity, suggesting that their antitumor effect is due to cellular mechanisms independent from the regulation of neuronal excitability [10, 11]. The epidemiological data coupled with the animal studies brought a newfound attention to CPZ as a potential anti-tumor medication, leading to follow-up studies focused on CPZ’s antitumor mechanisms. Studies in the past few decades have found that CPZ inhibits cancer growth through multiple independent pathways, via remarkably diverse targets ranging from histone deacetylases to ion channels. Here, we review the proposed molecular mechanisms of CPZ’s anti-tumor activity and cancer type specificity, and discuss the clinical potential of CPZ for cancer treatment.

## CHLORPROMAZINE AS AN ANTIPSYCHOTIC MEDICATION

**Figure 1:**
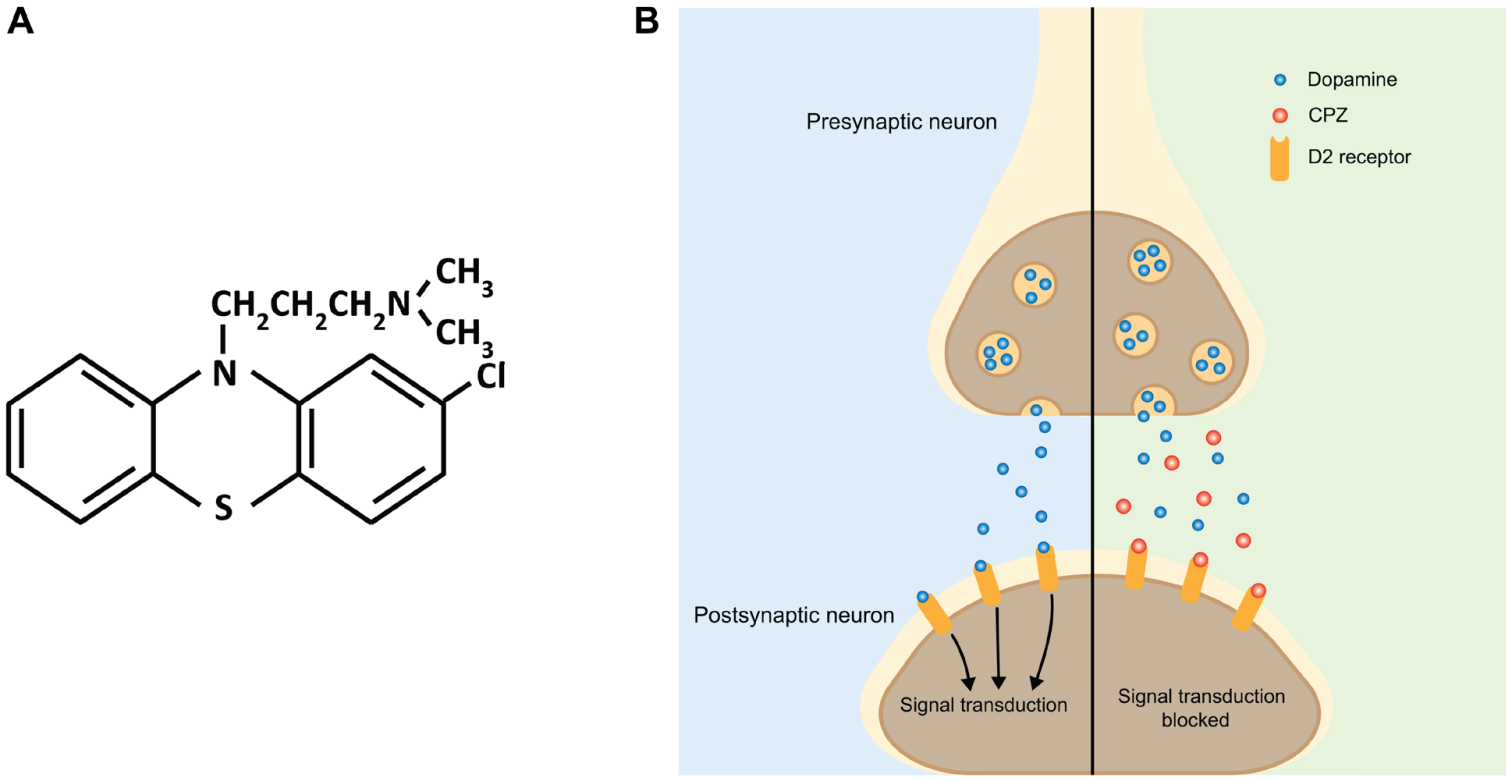
Schematic of the effect of CPZ on dopaminergic neurotransmission. (**A**) Chemical structure of CPZ. (**B**) In the absence of CPZ, release of dopamine from presynaptic terminals activates postsynaptic dopamine receptors, including D2, initiating downstream signal transduction (left side, blue). CPZ binds to D2 receptors without activating them. This prevents dopamine from binding to D2 receptors and blocks downstream signal transduction (right side, green).

CPZ, first synthesized by Paul Charpentier in 1951, was initially used as an anesthetic (structure shown in Figure 1A) [12]. CPZ’s psychiatric relevance was discovered in 1952 when its administration as an anesthetic during surgery resulted in ‘disinterest’ in patients [1]. Subsequent studies showed that CPZ can calm severely agitated psychotic patients, paving the way for its use as an antipsychotic medication worldwide [13]. Within the first decade of CPZ’s discovery, more than 50 million people worldwide had taken the drug [14]. CPZ remains one of the most common drugs used for treatment of psychiatric disorders and is designated as an essential drug for treatment of schizophrenia by the World Health Organization.

As the use of CPZ for treatment of psychiatric disorders increased in popularity, identification of its pharmacological mechanisms gained interest as well. Initially, it was thought that the antipsychotic effect of CPZ was mediated through the inhibition of serotonin receptors [15]. However, subsequent studies established a model still accepted today, in which inhibition of D2 dopamine receptors is primarily responsible for CPZ’s antipsychotic effect (Figure 1B) [16–18]. CPZ shows high specificity for dopamine receptors with a binding affinity of 3–23 nM for D2 dopamine receptors [19, 20]. Subsequent studies showed that CPZ and related drugs prevent dopamine from binding and activating D2 dopamine receptors in the areas of the brain involved in the control of emotional behavior, such as the limbic system [21, 22]. The antagonistic action of CPZ on D2 receptors alleviates psychotic symptoms associated with increased dopamine signaling found in psychiatric disorders, including schizophrenia, bipolar disorder and psychotic disorders [23].

The effectiveness of CPZ as an antipsychotic drug stems from its ability to readily permeate the blood brain barrier (BBB) with an apparent permeability of 303 nm/s [24, 25]. The high BBB permeability of CPZ is conferred by its low molecular weight (318.86 g/mol), absence of hydrogen bond donors, and low flexibility of its chemical structure (presence of rotatable bonds) - properties typical of successful Central Nervous System (CNS) drugs [25]. CPZ’s effectiveness as a CNS drug is further potentiated by its ability to remain in the brain for sufficient periods to exert its intended effect, with a half-life of 8 to 35 hours [26]. Typical oral doses of CPZ range from 100 to 1000 mg/day, depending on the severity of the psychiatric ailment [27]. The plasma levels of CPZ normally observed in patients are in the range of 0.1 μM to 2 μM [28, 29]. However, due to the high membrane permeability and tissue accumulation of phenothiazines, the effective cellular concentration of CPZ is expected to be 10 to 1000-fold higher [30–32].

## EARLY REPORTS OF CPZ’S CELLULAR ANTITUMORIGENIC MECHANISMS

The wide use of CPZ as an antipsychotic medication prompted studies on its effect on a wide range of cellular targets. It became clear that in addition to the neuronal receptors, CPZ also affects multiple seemingly unrelated targets and cellular processes, including inhibition of DNA synthesis [33], uncoupling of oxidative phosphorylation and inhibition of cytochrome oxidase [34, 35], suppression of adenosine triphosphatase (ATPase) enzymatic activity [34, 36], alteration of membrane permeability [37, 38] and inhibition of lipase activity [39]. Many of the identified cellular mechanisms were either initially or eventually linked to CPZ’s anticancer activity. These early studies indicated that while CPZ’s antipsychotic mechanism is relatively straightforward, its anticancer effect involves a diverse range of cellular pathways. During the last few decades, the number of targets and pathways implicated in CPZ’s anticancer activity has continued to grow, strengthening the potential of repurposing CPZ for cancer treatment. Below, we discuss some of CPZ’s most prominent cellular targets and mechanisms of action related to cancer.

## EFFECT OF CPZ ON DNA SYNTHESIS

**Figure 2:**
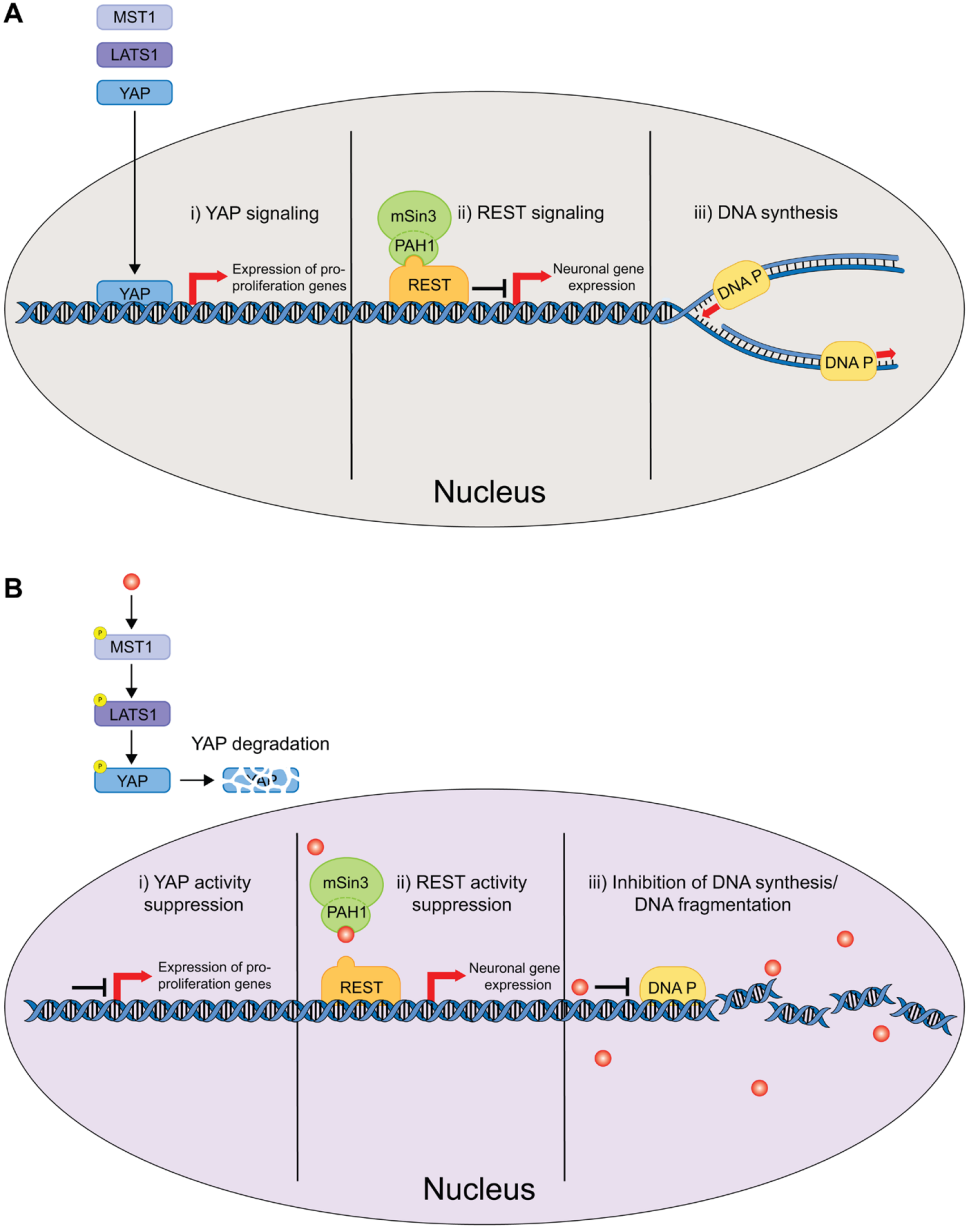
Schematic of the nucleus delimited effects of CPZ. (**A**) In the absence of CPZ, YAP signaling upregulates expression of genes involved in cell proliferation (i), REST signaling activates neuronal gene expression (ii) and DNA Polymerase carries out DNA replication necessary for cell division (iii). (**B**) CPZ inhibits YAP signaling by inducing YAP degradation (i), inhibits REST signaling most likely by binding to PAH1 and inhibiting mSin3/REST interactions (ii), and inhibits DNA Polymerase and causes DNA fragmentation (iii). These effects of CPZ inhibit cell proliferation and tumorigenesis.

Cancer is characterized by an uncontrolled proliferation of cells. DNA synthesis is crucial to cell division, thus, inhibition of DNA synthesis may bridle cancer progression (Figure 2A). DNA synthesis inhibition was amongst the first identified CPZ’s anticancer cellular mechanisms. In 1965, it was shown that CPZ decreases H^3^-thymidine incorporation into human bone marrow cells during DNA synthesis, indicating that CPZ inhibits DNA synthesis (Figure 2B (iii)) [40, 41]. Similarly, inhibition of DNA synthesis was observed in Meth A sarcoma cells [42]. The inhibition of DNA synthesis by CPZ was reported in cell culture and cell-free systems [33, 42], suggesting that CPZ could be directly inhibiting enzymes involved in DNA synthesis. In addition to inhibiting DNA synthesis, CPZ caused DNA fragmentation in leukemic and mouse mastocytoma cells [43, 44], and in human oral cancer cells via the inhibition of Akt/mTOR phosphorylation (Figure 2B (iii) (see below) [45]. Importantly, CPZ decreased the viability of leukemic cells with minimal cytotoxicity and at clinically relevant doses (1–40 μM range) [43]. Similarly, while CPZ inhibited oral cancer cell proliferation with the IC50 of ~20 μM, it had little effect on healthy (noncancerous) oral cells [45]. CPZ has also been shown to inhibit the initial step of SV40 DNA replication in HeLa cell extract, specifically the pre-elongation step [46]. Taken together, these studies demonstrate that CPZ can inhibit cell proliferation in cancer by modulating DNA synthesis, although further studies are needed to tease out the direct mechanisms involved.

## EFFECT OF CPZ ON REST MEDIATED GENE TRANSCRIPTION

Repressor-element 1 silencing transcription factor (REST), also known as neural restrictive silencer factor (NRSF), represses neuronal gene transcription in non-neuronal cells, and regulates neuronal differentiation and gene expression (Figure 2A (ii)) [47–49]. REST represses gene expression by recruiting co-repressors mammalian Swi-independent 3 (mSin3) and CoREST1 [50–52]. The co-repressor mSin3 contains four paired amphipathic helical (PAH) domains, PAH1-PAH4, with PAH1 binding to REST [53, 54]. Importantly, upregulation of REST expression has been linked to several brain tumors, including medulloblastoma and glioblastoma [55–58].

In 2018, Kurita and coworkers identified CPZ as a small-molecule binder of mSin3 PAH1, using a ligand-based and structure based *in-silico* drug screening [59]. NMR titration experiments indicated that CPZ binds to the isolated PAH1 fused to Glutathione S-transferases (GST) with the binding affinity of 15 ± 4.2 μM. NMR data-guided docking of CPZ into PAH1 structure indicated CPZ-induced structural rearrangements. It has been proposed that by binding to PAH1 and inducing structural changes, CPZ could block mSin3-REST interactions, thereby reducing REST mediated transcription repression (Figure 2B (ii)). Consistent with this hypothesis, application of CPZ to human DAOY medulloblastoma cells decreased spheroid growth [59]. Although other mechanisms of the CPZ action have not been ruled out in the study, destabilization of mSin3-REST interactions could be yet another mechanism contributing to tumor growth inhibition by CPZ.

## EFFECT OF CPZ ON YAP SIGNALING

Yes-associated protein (YAP) is a component of the Hippo signaling pathway and promotes cell proliferation and organ growth when it is not phosphorylated (Figure 2A (i)) [60–62]. Activation of the Hippo signaling cascade results in the phosphorylation of large tumor suppressor kinase 1/2 (LATS1/2) by the mammalian Ste2-like kinase (MST1/2). LATS1/2 in turn phosphorylates YAP, which leads to YAP proteasomal degradation. Recently, YAP overexpression was found to be associated with liver [63] and breast cancers [64, 65]. Moreover, increased YAP expression was shown to confer cancer cells with stem-like properties, including chemoresistance [66, 67]. Cancer stem cells (CSC) are one of the most intractable obstacles for cancer treatment due to their self-renewal properties and the ability to differentiate, leading to multidrug resistance and tumor recurrence. Importantly, YAP inhibitors have been shown to suppress CSC properties, such as drug resistance [67–69].

In 2019, Yang and coworkers found that CPZ suppresses YAP signaling in MCF7 and MDA-MB-231 breast cancer cells [70]. CPZ treatment induced a dose-dependent increase in the phosphorylation of YAP’s upstream regulators, LATS1 and MST1, thereby promoting YAP phosphorylation and subsequent proteasomal degradation, and decreased nuclear expression levels of YAP (Figure 2B (i)). Importantly, inhibition of YAP signaling by CPZ suppressed stemness in breast cancer cells, causing decrease in self-renewal and chemoresistance to doxorubicin and taxol, commonly used for breast cancer chemotherapy. In summary, the effect of CPZ on YAP and upstream Hippo signaling could be an important pathway for decreasing stemness and increasing the effectiveness of existing breast cancer chemotherapies.

## EFFECT OF CPZ ON MITOTIC KINESIN

**Figure 3:**
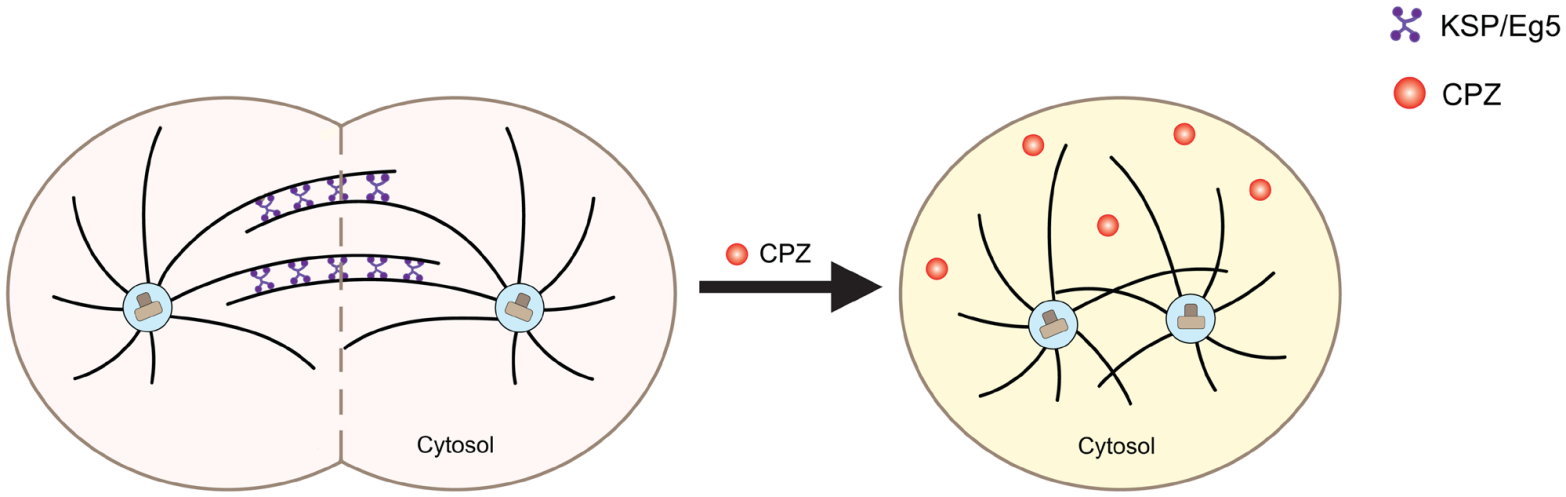
Schematic of the effect of CPZ on mitotic kinesins. Mitotic kinesins, such as KSP/Eg5, are crucial for mitotic spindle assembly for chromosome segregation during mitosis (left, pink). CPZ is thought to inhibit mitotic kinesins, hindering the spindle assembly and increasing the number of cells with monopolar spindles (right, yellow).

Mitosis is a crucial phase in the cell cycle and, therefore, is an important target for cancer therapies. Recent efforts to target mitosis for cancer treatment focus on suppressing cell division via affecting mitotic kinesins [71]. Mitotic kinesins are a family of mechanochemical enzymes that use ATP to traverse along or destabilize microtubules. These proteins are crucial for cell division and regulate the function of mitotic spindle (Figure 3, left) [72, 73]. One member of the mitotic kinesin family, KSP/Eg5, has been considered a promising oncogenic target. Substantial effort has been levied to identify KSP/Eg5 inhibitors for therapeutic use [74].

In 2007, Lee and coworkers showed that CPZ inhibited KSP/Eg5’s ability to hydrolyze ATP with an IC50 of 5 to 10 μM [75]. The effect was specific to KSP/Eg5, as no CPZ-dependent inhibition was observed for any other mitotic kinesins or chromokinesins, even at CPZ concentrations as high as 100 μM. Treatment with CPZ inhibited proliferation of HCT116 human colon carcinoma cell lines with an IC50 of 5 to 7 μM. Moreover, application of CPZ in combination with pentamidine, an antiparasitic agent, inhibited tumor growth in mice xenograft models implanted with A549, non–small cell lung cancer (NSCLC), or HCT116 colon cancer cells. At the cellular level, CPZ treatment increased the population of cells with monopolar spindle and the population of cells in the G2-M phase of cell cycle. Both accumulation of monopolar spindles and cell cycle arrest are characteristic of KSP/Eg5 inhibition, thereby suggesting that CPZ inhibits KSP/Eg5 activity, and this inhibition is involved in the observed antitumorigenic effect (Figure 3, right). Application of other phenothiazines with chemical structures similar to CPZ caused a similar antitumorigenic effect, although, with lower potency, further suggesting that the inhibition of KSP/EG5 activity is contributing to the reduction in cancer growth. Consistent with these results, Riffell and coworkers reported that CPZ treatment caused mitotic arrest in MCF-7mp53 breast cancer cells expressing dominant-negative p53, MDA-MB-231 breast cancer cells and T98G glioblastoma cells [76]. CPZ induces mitotic arrest at concentrations higher than 10 μM when used alone, however, it was effective at lower concentrations when administered in combination with 10–30 nM paclitaxel, a microtubule-targeting drug, suggesting the drugs work synergistically. Taken together, these studies indicate that inhibition of mitotic kinesin activity by CPZ can suppress tumor growth by inducing cell cycle arrest due to an accumulation of cells with monopolar spindle. Therefore, mitotic kinesin inhibition is yet another mechanism contributing to CPZ’s antitumorigenic effect.

## EFFECT OF CPZ ON CYTOCHROME C OXIDASE

**Figure 4:**
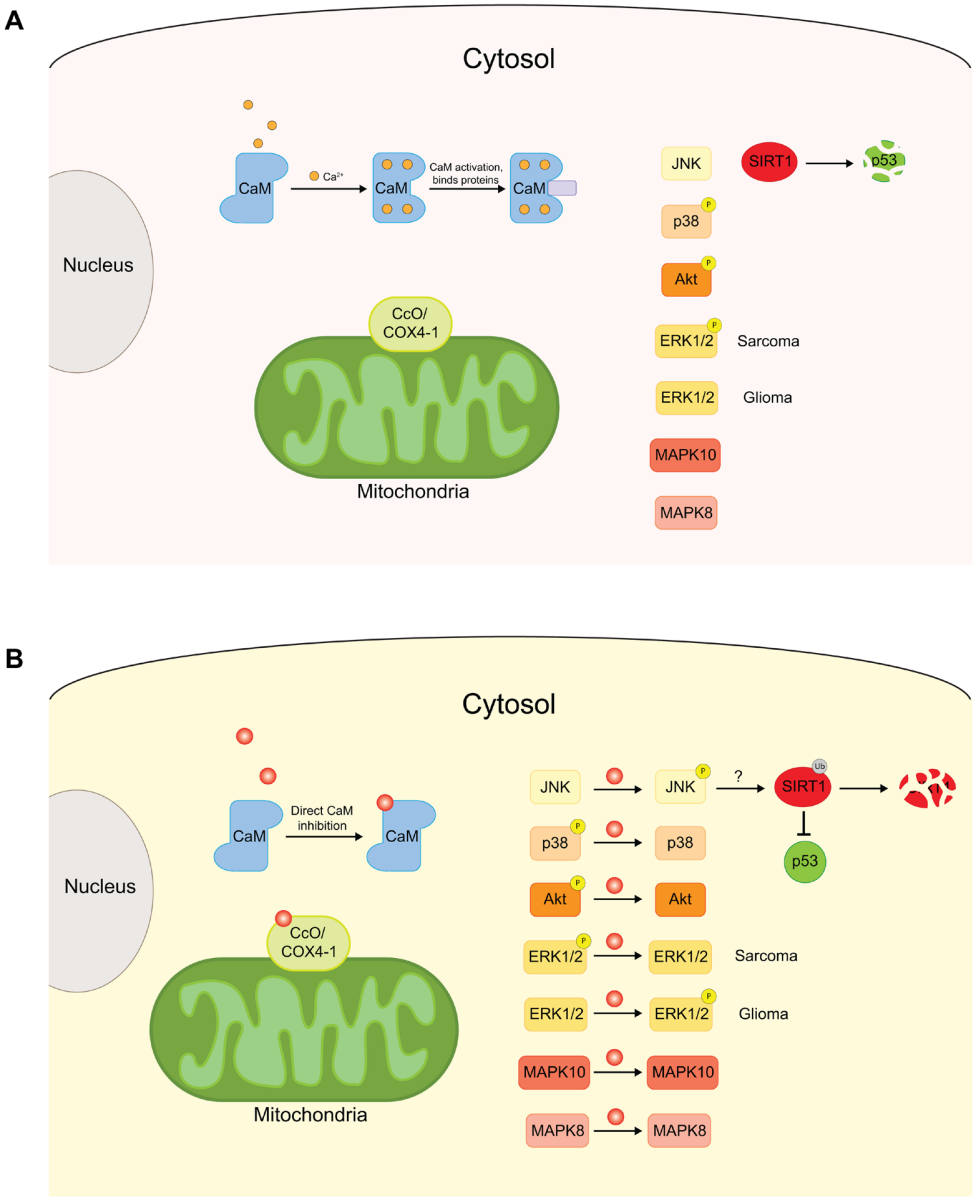
Schematic of the effects of CPZ on cytoplasmic and mitochondrial proteins. (**A**) Cytoplasmic proteins, CaM, kinases, including JNK, p38, Akt, ERK1/2 and MAPKs, and mitochondrial CcO complex are important proteins for normal cellular functions. Defects in these proteins can increase cell proliferation and drive tumorigenesis. (**B**) CPZ has been shown to inhibit CaM, affect the phosphorylation levels of the kinases, and preferentially bind to COX4-1 subunit, enriched in CcO complexes associated with cancer. Through these effects, CPZ inhibits cell proliferation and tumor progression.

Cytochrome c oxidase (CcO) is the final enzyme of the mitochondrial electron transport chain (Figure 4A). CcO catalyzes the transfer of electrons from cytochrome c to oxygen (O_2_) molecules and subsequently converts O_2_ to water. In mammals, CcO is composed of thirteen subunits, many of which have multiple isoforms [77]. Several studies have identified links between CcO and tumor development and progression. It has been shown that the various CcO subunits are differentially expressed in certain tumors. For instance, expression of COX3 subunit of CcO is lower than normal in colon adenomas [78], while COX4 subunit isoform 1 (COX4-1) is overexpressed in glioblastoma (GBM) cells, and its upregulation is linked to the development of temozolomide (TMZ) chemoresistance in GBM cells [79]. Differential expression of COX5b subunit has been reported in colorectal carcinomas [80], and knockdown of the COX5a subunit by siRNA has been shown to reduce non-small cell lung cancer (NSCLC) cell migration and invasion [81].

Early studies of the effects of CPZ on CcO were conducted in mid 1950s on the rat brain and liver mitochondria [30, 32]. It was found that CPZ inhibits CcO activity by 50% at 50 μM concentration in rat brain mitochondria [34]. The direct link of CcO activity inhibition by CPZ to cancer was investigated in a recent study by Oliva and coworkers [82]. It was found that CPZ inhibits CcO activity in a dose-dependent manner in TMZ resistant U251 cell-derived UTMZ glioma cells while having no effect on TMZ-sensitive U251 cells. The specificity of the CcO inhibition was proposed to stem from a preferential binding of CPZ to a binding site at the interface of COX4-1 and two other subunits of the CcO complex (Figure 4B). Since the expression of COX4-1 subunits is increased in TMZ-resistant glioma cells, the preferential binding of CPZ to COX4-1 would explain the specificity of the CPZ effect for the TMZ-resistant U251 glioma cells [79]. Taken together with the previous reports, the study by Oliva and coworkers indicates that the inhibition of CcO activity by CPZ is one of the contributing factors to the CPZ’s antitumorigenic activity.

## EFFECT OF CPZ ON KINASES

Mitogen-activated protein kinases (MAPK) are protein kinases that are involved in directing cellular responses to a diverse range of signals, including mitogens, osmotic stress, pro-inflammatory cytokines, and growth factors (Figure 4A) [83]. MAPK signaling pathways play an important role in cell proliferation and differentiation and are frequently dysregulated in cancer [83, 84]. Recent bioinformatics study based on regulation network analysis of protein interactions identified MAPK8 and MAPK10 as potential targets of CPZ (Figure 4B) [85]. Experimental findings further substantiated the possibility of MAPK contributing to the antitumor effects of CPZ. In 2010, Shin and coworkers showed that CPZ induced phosphorylation of extracellular signal-regulated kinase 1/2 (ERK1/2) and c-Jun N-terminal kinase (JNK), members of the MAPK family, in C6 glioma cells [86]. The increased phosphorylation of ERK1/2 and JNK kinases was linked to the induction of cyclin-dependent kinase inhibitor 1 (CDKN1), also known as p21 or p21^Waf1/Cip1^, by the transcription factor early growth response-1 (Egr-1), which itself is transcriptionally activated by another transcription factor, the ternary complex factor also known as Elk-1 [87]. CDKN1 promotes cell cycle arrest at the G1 and G2/M phases [88–90] and mediates the ability of the tumor suppressor p53 to arrest cell proliferation [91]. The upregulation of the JNK(ERK1/2)/Elk-1/Egr-1/p21 pathway contributed to the antitumorigenic effect of CPZ in C6 glioma cells, as summarized in Figure 4B) [86].

While CPZ increased JNK and ERK1/2 phosphorylation in C6 cells, the same study demonstrated that CPZ decreased p38 MAPK phosphorylation in the same cell line [86]. This suggests that the effect of CPZ on kinases is diverse and could be different even for the same kinases depending on the cell-type. In agreement with the varied effect of CPZ on MAPK phosphorylation, Martins and coworkers showed that CPZ decreases phosphorylation of ERK1/2 in Ewing sarcoma (ES) cells leading to reduction in cell proliferation and increase in the cell apoptosis levels [92], whereas Lee and coworkers showed that CPZ increased JNK phosphorylation in human colorectal cancer HCT116 cells, causing inhibition of the tumor cell growth and increased apoptosis [93]. Further investigation indicated that CPZ-induced JNK phosphorylation caused degradation of p53 inhibitor sirtuin 1 (SIRT1), either directly or indirectly, thereby inducing p53-dependent apoptosis of HCT116 cells (Figure 4B) [93]. Taken together, the reported effects of CPZ on the MAPK family are overwhelmingly antitumorigenic, however, the direction of the effect varied with tissue- and kinase-type.

Another kinase affected by CPZ is Akt, also known as protein kinase B, which in turn activates the mammalian target of rapamycin (mTOR). Akt/mTOR pathway regulates cell cycle and the induction of autophagy, and deficiencies in the pathway have been linked to cancer [94, 95]. Recently, Shin and coworkers found that CPZ, either directly or via intermediate players, inhibited mTOR by decreasing the levels of phosphorylated Akt (Figure 4B) [96]. CPZ inhibited the Akt/mTOR pathway in PTEN (phosphatase and tensin homolog)-null U-87MG glioma cells, with constitutively active Akt/mTOR pathway, causing cell cycle arrest and autophagic cell death. Similarly, Jhou and coworkers found that in oral cancer, CPZ reduced levels of phosphorylation of Akt and mTOR, leading to the cell cycle arrest in G2/M phase and inhibition of cell proliferation [45]. Despite the evidence that the kinases are involved in the effect of CPZ in cancer, experimental proof of direct binding of CPZ to kinases is lacking. Therefore, the molecular mechanisms of CPZ action on the kinases are not clear and require further investigation.

## EFFECT OF CPZ ON CALMODULIN

Calmodulin (CaM) is a multifunctional calcium-binding protein ubiquitously expressed in eukaryotic cells (Figure 4A). CaM interacts with many other proteins to mediate a diverse range of cellular functions and signaling pathways. Amongst CaM’s main functions are the regulation of cell proliferation, division and differentiation [97], all of which contribute to its role in tumorigenesis. CaM was found to be upregulated in human primary lung cancer cells [98], and in rat fibroblasts transfected with oncogenes [99]. Moreover, CaM antagonists have been reported to decrease lung metastasis induced by implanting Lewis lung carcinomas in mice [100] and decrease tumor cell growth in different cancer types [101–103].

CPZ is known to inhibit CaM activity by direct binding to the protein in a calcium-dependent manner (Figure 4B) [104]. In 1982, Prozialeck and Weiss reported that the hydrophobicity of the phenothiazine ring of CPZ and other phenothiazine antipsychotics correlates well with their potency in inhibiting CaM activity, as reflected in the inhibition of CaM-induced activation of phosphodiesterases [105]. The authors proposed that CPZ binding to CaM is mediated by hydrophobic interactions between the phenothiazine ring and a nonpolar region of calmodulin, and by an electrostatic interaction between the positively charged amino group of CPZ and negatively charged residues on CaM protein. In the same study, the IC50 for CaM activity inhibition by CPZ was reported to be 40 μM.

Although several CaM inhibitors have successfully quelled cancer growth and metastasis, it is not clear if CaM inhibition by CPZ is therapeutically relevant. For instance, Zhong and coworkers reported that CPZ has antitumorigenic effect on small cell lung carcinoma (SCLC) [106]. However, application of CaM inhibitor W7 failed to reproduce the effect of CPZ, indicating that the CPZ’s antitumorigenic activity in SCLC is independent of CaM. Therefore, although it would be reasonable to speculate that inhibition of CaM activity by CPZ could be yet another mechanism contributing to CPZ’s antitumorigenic effect, evidence substantiating this hypothesis has yet to be found.

## EFFECT OF CPZ ON MEMBRANES

**Figure 5:**
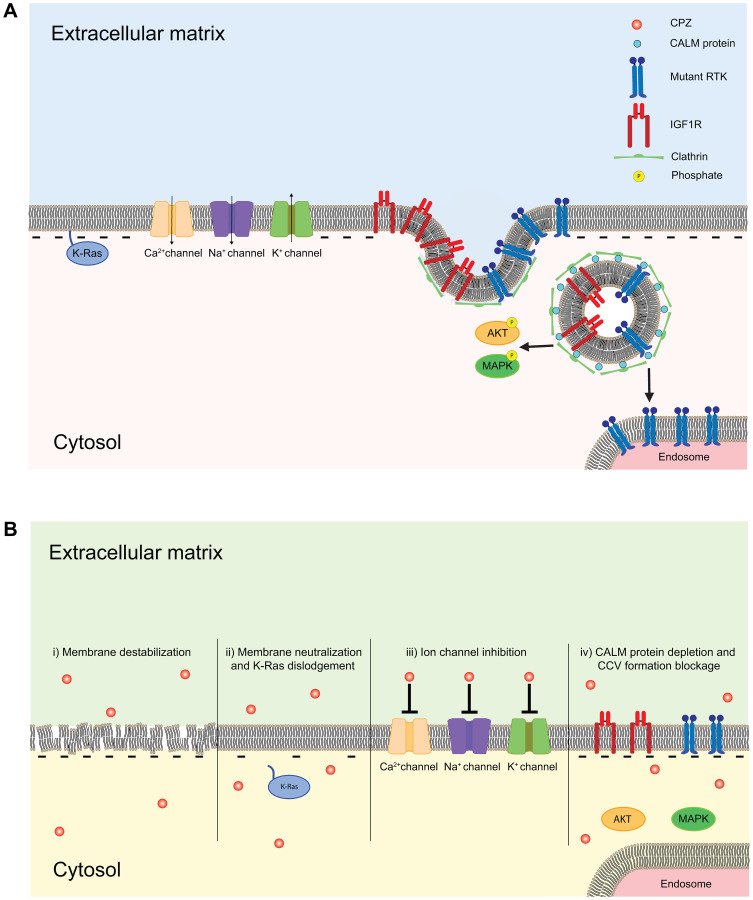
Schematic of the membrane delimited effects of CPZ. (**A**) Cellular surface membrane is formed by lipids, and contains transmembrane and membrane-associated proteins. Transmembrane proteins such as ion channels, including EAG, ERG and other K^+^ channels, Na^+^ channels and Ca^2+^ channels, receptors, including RTKs and IGF1Rs, and membrane-associated proteins, including Ras proteins, are key regulators of cell cycle and tumorigenesis. (**B**) CPZ directly affects membrane by destabilizing the lipid bilayer and increasing membrane permeability (i), decreasing membrane association of Ras protein by neutralizing the negative charge of phospholipids (ii), inhibiting various ion channels, including K^+^, Na^+^ and Ca^2+^ selective channels (iii) and altering RTK and IGF1R receptor internalization by suppressing clathrin mediated signaling (iv).

Cellular membranes are vital for cellular integrity and contain numerous transmembrane proteins essential for cellular signaling (Figure 5A). An effect of CPZ on membranes was first reported in the 1960s when it was shown that CPZ inhibited the swelling of rat liver mitochondrial membranes in isotonic sucrose solution [37], reduced the rate of hypotonic hemolysis in human erythrocytes [107], and inhibited tissue uptake of radioactively labeled norepinephrine [108, 109]. It was suggested that these effects were due to the CPZ-induced decrease in membrane permeability, yet the exact mechanism was not elucidated. On the other hand, *in vivo* and *in vitro* studies of effects of CPZ on rat liver lysosomes suggested that at high concentrations (>100 μM) CPZ increases membrane permeability [38]. The discrepancy in the effect of CPZ on cellular membranes reported in the early studies could reflect the difference in the experimental systems used or could indicate that CPZ’s effect is dependent on membrane composition.

More recent studies showed that application of CPZ causes leakage of chromophore carboxyfluorescein from liposomes [110], and leakage of [^18^F]2-fluoro-2-deoxy-D-glucose-6-phosphate tracer and increased membrane fluidity in rat brain slices (Figure 5B (i)) [111]. The latter effect of CPZ was observed only at concentrations >100 μM, which are higher than the putative therapeutically relevant plasma levels of CPZ. However, due to the 20- to 30-fold accumulation of antipsychotic drugs in brain tissues [30, 31] and further accumulation in cellular membranes due to their high lipophilicity, the effect of CPZ on the membrane permeability and stability could be therapeutically important. In 2009, CPZ’s effect on membrane permeability was further characterized in membrane vesicles, where CPZ drastically increased membrane permeability as reflected in the CPZ concentration-dependent release of fluorophor calcein [112], and lipid monolayers, where CPZ increased the surface area of monolayers formed from acidic phospholipids while having no effect on monolayers formed from neutral lipids [113]. Investigation of interactions between CPZ and model membranes with the differential scanning calorimetry and isothermal calorimetry suggested that CPZ both interacts with negatively charged membrane phospholipids and also intercalates into the hydrophobic region of the membrane bilayer [112]. The interaction between CPZ and the membrane resulted in the increased membrane permeability and overall destabilization of the membrane, possibly by introducing membrane pores (Figure 5B (i)). Consistent with this hypothesis, CPZ-induced increase in membrane permeability resulted in increased MCF-7 human breast cancer cells sensitivity to tamoxifen, demonstrated by the prevention of cell growth and reduction of metabolic activity in the cells [112]. Therefore, CPZ-induced increase in membrane permeability facilitated increased accumulation of tamoxifen in the MCF-7 cancer cells, thereby increasing the drug’s efficacy. Taken together, these recent studies demonstrate that CPZ induces increased membrane permeability, thereby increasing intracellular accumulation of anticancer drugs and ultimately enhancing their effectiveness.

In addition to its effect on membrane permeability, CPZ has been shown to alter membrane polarity by interacting with negatively charged membrane phospholipids, in both live cells and phospholipid bilayers (Figure 5B (ii)). Confocal microscopy and fluorescence recovery after photobleaching (FRAP)-based studies found that CPZ facilitated the membrane dislodgment of oncogenic K-Ras4B(G12V), an isoform of Ras protein that anchors to the membrane via electrostatic interaction [114]. Ras proteins are membrane-associated GTPases, cycling between inactive GDP-bound and active GTP-bound states. Ras proteins are important regulators of cell growth and proliferation, and about 30% of human cancers are associated with defects in Ras protein function [115]. Proper anchorage of Ras proteins to the membrane is essential for their role in cellular signaling and tumorigenesis. CPZ significantly increased cytoplasmic and/or mitochondrial levels of K-Ras4B(G12V), while decreasing K-Ras4B(G12V) levels in the membrane. Importantly, CPZ did not have an effect on H-Ras, a Ras protein isoform which does not require electrostatic interaction with negatively charged phospholipids for its association with the membrane. This suggests that the CPZ dislodges K-Ras4B(G12V) by neutralizing negatively charged membrane phospholipids, thereby weakening the protein’s electrostatic interaction with the membrane (Figure 5B (ii)). Importantly, CPZ-induced membrane dislodgement of K-Ras4B(G12V) was associated with apoptosis of Rat-1 fibroblasts cell lines stably expressing GFP-K-Ras(G12V), and cell cycle arrest and suppressed wound healing in human pancreatic cancer PANC-1 cell lines expressing GFP-K-Ras(G12V) cells [114]. Consistent with CPZ-induced membrane dislodgment of K-Ras4B(G12V) via affecting membrane phospholipids, fluorescence spectroscopy studies indicated that CPZ increased disorder of the polar heads and acyl chain regions of the phospholipid bilayer, destabilizing the membrane [116]. In summary, the studies on the effects of CPZ on cellular membranes indicate that it affects both membrane permeability and polarity, and both effects contribute to CPZ’s antitumorigenic activity, as summarized in Figure 5B. The diverse range of CPZ’s effects on cellular membranes further underscores the complexity of its mechanism.

## EFFECT OF CPZ ON ION CHANNELS

The initial reports of CPZ effects on ion channels came from studies of calcium channels. It was shown that CPZ and other antipsychotic drugs inhibit the binding of [^3^H]nitrendipine, a calcium channel antagonist, to calcium channels with an affinity comparable to CPZ’s affinity for dopamine receptors [117], causing the inhibition of smooth muscle contractions [117, 118]. Subsequent studies found that CPZ blocks a variety of sodium, potassium, and calcium channels (Figure 5B (iii)) [119–123].

More recently, it was shown that CPZ inhibits ether-a`-go-go (EAG) and EAG-related gene (ERG) channels, which belong to the KCNH family of potassium selective channels [124–126]. EAG and ERG are considered bona fide oncogenic channels [127, 128]. Upregulation of EAG and ERG channel activity has been implicated in the proliferation and progression of various cancers [129–137]. Additionally, the overexpression of EAG channels in human tumors is clinically used as tumor marker [129, 138, 139]. Inhibition of EAG and ERG channel activity, either with non-specific blockers or by siRNA, has suppressed cancer growth [137, 140–142]. It was shown that CPZ inhibits hERG channels in a concentration and voltage-dependent manner with the IC50 of 10.5 μM at -30 mV, and 4.9 μM at +30 mV, most likely by blocking channel pore [125, 126]. More recently, we found that CPZ also inhibits EAG channel activity [124], however, the mechanism of inhibition seems to be different from ERG channel inhibition. CPZ inhibited EAG currents in a concentration-dependent and voltage-independent manner with the IC50 of 3.7 μM [124]. Our study suggests that CPZ inhibits EAG channels by binding to the intracellular Per-Arnt-Sim (PAS) domain of the channels, as fluorescence-based and surface plasmon resonance-based assays showed direct binding of CPZ to the PAS domain and deletion of the PAS domain dramatically decreased the CPZ inhibition of EAG currents. Interestingly, we found that CPZ does not bind to the PAS domain of ERG channels. Taken together, the studies of CPZ’s effect on oncogenic EAG and ERG channels suggest that inhibition of these channels by CPZ likely contributes to its antitumorigenic effect.

## EFFECT OF CPZ ON CALM PROTEIN

Clathrin assembly lymphoid myeloid leukemia (CALM) protein is crucial for the formation of clathrin coated vesicles for intracellular trafficking of receptor tyrosine kinases (RTKs) [143]. Mutations in RTKs are linked to various cancers, including lung cancer and acute myeloid leukemia (AML) [144]. While wild-type RTKs are continuously shuffled between the plasma membrane and endosomes via clathrin-mediated internalization and subsequent recycling, mutant RTKs are mistargeted to different intracellular compartments, such as endosomes, where they activate downstream targets and promote oncogenic activity [145, 146]. Recently, CPZ was shown to reduce the levels of CALM protein in a concentration-dependent manner, thereby altering the cellular localization of RTKs with AML-associated mutations, without disturbing wild-type RTKs (Figure 5B (iv)) [147]. Both treatment with CPZ and CALM knock-out resulted in the dislocation of the mutant RTKs and decreased levels of phosphorylation of downstream targets of RTKs. Also, both CPZ treatment and CALM knock-out inhibited the growth of AML cells with mutant RTKs. Importantly, CPZ treatment had no effect on the growth rate of AML cells with CALM knock-out, implicating suppression of CALM protein levels as the primary mechanism of CPZ’s anticancer effect in AML cells expressing mutant RTKs. In summary, the study provides evidence that CPZ is capable of blocking compartment-dependent oncogenic activity of mutant RTKs, via inhibition of the CALM protein.

## ANTITUMORIGENIC ACTIVITY OF CPZ IN DIFFERENT CANCERS

In this section we list known antitumorigenic CPZ effects in different cancers and reported/proposed cellular mechanisms (Table 1). In breast cancer, CPZ exhibits an anti-proliferative effect, suppresses stemness, and increases cancer cell sensitivity to existing chemotherapies, thereby reversing drug resistance [70, 112]. The implicated mechanisms of CPZ action in breast cancer are suppression of YAP signaling [70] and increase in membrane permeability that promotes accumulation of chemotherapeutic agents, including doxorubicin and taxol [70]. In colorectal cancer, CPZ was shown to inhibit tumor growth and induce apoptosis [93]. The examined effect of CPZ in colorectal cancer was attributed to a down-regulation of p53 inhibitor, SIRT1. Interestingly, YAP levels promote colorectal tumor aggressiveness [148]. Therefore, similar to breast cancer, YAP suppression by CPZ in colorectal cancer could be in part responsible for its observed antioncogenic effect.

**Table 1:** Summary of antitumorigenic effects of CPZ in different cancers

Cancer type	Effective concentration	Proposed Mechanism	Cell line (human, unless indicated)	Ref
Breast cancer	10 μM	Increase in membrane permeability	MCF-7	[112]
	> 2 μM	Suppression of YAP signaling	MCF-7, MDA-MB-231	[70]
	≥6 μM	Inhibition of KSP/Eg5	MCF-7mp53, MDA-MB-231	[76]
Colorectal cancer	3–10 μM (IC50)	SIRT1 inhibition	HCT116 LoVo	[93]
	5–7 μM (IC50)	Inhibition of KSP/Eg5	HCT116	[75]
Brain tumor	4.5 μM (IC50)	Destabilization of REST-mSin3 interaction	DAOY (medulloblastoma)	[59]
	13 μM (IC50)	COX4-1 inhibition	TMZ-resistant U251 (glioblastoma)	[82]
	20–40 μM	Upregulation of the JNK(ERK1/2)/Elk-1/Egr-1/p21 pathway	C6 (glioma)	[86]
	>20 μM	Inhibition of Akt/mTOR pathway	PTEN and U-87MG glioma cells	[96]
	≥6 μM	Inhibition of KSP/Eg5	T98G (glioblastoma)	[76]
	Not known	Not known	RG2 (rat glioblastoma)	[151]
Skin cancer	36.6 μM (IC50)	Not known	B16 mouse melanoma	[149, 150]
	12–19 mg/kg/day (drug dose used in mouse model)	Not known	Harding-Passey Melanoma mouse melanoma	[150]
Leukemia	12.04 μmol/l (IC50)	Inhibition of mitochondrial DNA polymerase and decreased ATP production	K-562 (chronic myelogenous leukemia)	[43]
	11.19 μmol/l (IC50)	Same as above	BALL-1 (B-acute lymphoblastic leukemia)	[43]
	6.57 μmol/l (IC50)	Same as above	MOLT-4 (T-acute lymphoblastic leukemia)	[43]
	11.81 μmol/l (IC50)	Same as above	CCRF-HSB-2 (T-acute lymphoblastic leukemia)	[43]
	12.33 μmol/l (IC50)	Same as above	HPB-ALL (T-acute lymphoblastic leukemia)	[43]
	6.940 μM (IC50)	Suppressed mutant RTK activity via CALM inhibition	Ba/F3/FLT3 ITD (modified murine pro-B cell line)	[147]
	6.942 μM (IC50)	Same as above	Ba/F3/ KIT D814V (modified mouse pro-B cell line)	[147]
Lymphoma	6.95 μmol/l (IC50)	Inhibition of mitochondrial DNA polymerase and decreased ATP production	Raji (Burkitt’s lymphoma)	[43]
	14.89 μmol/l (IC50)	Same as above	Daudi (Burkitt’s lymphoma)	[43]
Lung cancer	10 μM	lysosomal dysfunction	H69, H82, H592 and U-1285 (small cell lung carcinoma cell lines)	[106]
Sarcoma	25 μM (IC50)	Inhibition of DNA synthesis	Meth A cells	[42]
	10–15 μM (IC50)	Suppressed IGF1R internalization	A673, A4573, TC71 cells (ES cells)	[92]
Mastocytoma	~ 7 μM (IC50)	DNA fragmentation	PY815 (mouse mast cells)	[44]
Pancreatic cancer	25 μM	Dislodging K-Ras from plasma membrane	PANC-1 (pancreatic carcinoma)	[114]
Lung cancer	CPZ + pentamidine	Inhibition of KSP/Eg5	A549 (NSCLC)	[75]
Oral cancer	26.65 ± 1.1 μM (IC50)	Inhibition of Akt and mTOR phosphorylation	HSC-3	[45]
	23.49 ± 1.26 μM (IC50)	Same as above	Ca9-22	[45]

CPZ has been shown to exert antitumorigenic activity in several brain tumors. Medulloblastoma is the most common pediatric brain tumor frequently associated with overexpression of transcriptional repressor REST protein [55, 58]. CPZ inhibited growth of DAOY medulloblastoma cell spheroids, possibly by preventing interaction between REST and mSin3 necessary for REST mediated transcription suppression [59]. Another brain tumor with reported antitumorigenic effect of CPZ is GBM, one of the most lethal malignant brain tumors. A common chemotherapy for GBM involves treatment with temozolomide (TMZ). However, development of chemoresistance and CSC-promoted tumor recurrence present a substantial barrier for treatment of GBM. CPZ exhibited a potent cytotoxic effect on TMZ-resistant U251 human GBM cells with an IC_50_ of 13 μM, and also inhibited the cell neurosphere formation and anchorage-independent growth in soft agar [82]. This antitumorigenic effect of CPZ was specific to the TMZ-resistant GBM cells, as little growth inhibition was observed for TMZ sensitive U251 cells. The specificity was attributed to preferential binding of CPZ to the COX4-1 subunit of CcO enriched in the TMZ-resistant U251 cells, as discussed above. Notably, GBM has also been linked to the dysregulation of REST protein [56, 57]. Though it has not been examined, CPZ-induced suppression of REST signaling may be acting in parallel to the CcO inhibition shown to be responsible for the antitumorigenic effect of CPZ in GBM [82]. CPZ also inhibited growth of C6 glioma cells by upregulation of the JNK and ERK1/2 kinase phosphorylation [86].

CPZ suppresses proliferation and induces apoptosis in cultured lymphoma and leukemia cells via DNA fragmentation without affecting the viability of normal lymphocytes [43]. Inhibition of DNA polymerase and ATP production by CPZ was proposed as the most likely mechanism for this effect. CPZ also suppresses the growth of AML cells by inhibiting CALM protein involved in the formation of clathrin-coated vesicles and causing dislocation of the mutant RTKS, without disturbing wild-type RTKs [147]. Interestingly, suppression of clathrin-mediated endocytosis has been implicated in the antitumorigenic effect of CPZ in Ewing sarcoma (ES), a childhood cancer that occurs in bones or surrounding soft tissue, where CPZ was shown to disrupt clathrin-dependent internalization of the insulin-like growth factor 1 receptor (IGF1R), inhibiting AKT and MAPK phosphorylation and reducing ES cell proliferation [92]. Therefore, inhibition of clathrin-dependent signaling might be one of the conserved mechanisms of CPZ’s antitumorigenic effects.

CPZ decreased proliferation of oral cancer cells while showing low toxicity in normal oral cell lines [45]. This effect was attributed to the decreased phosphorylation of Akt and mTOR, as was the case in glioma cells [96]. CPZ inhibited proliferation of human colon carcinoma cells via inhibition of KSP/Eg5 mitotic kinesin [75], which was the same mechanisms responsible for the inhibition of MCF-7mp53 and MDA-MB-231 human breast cancer, and T98G glioblastoma cell lines [76]. Additionally, CPZ inhibited mouse mastocytoma cell growth by inducing DNA breakage [44], Meth A cell sarcoma growth via DNA synthesis inhibition [42], lung cancer growth by inducing lysosomal dysfunction [106], and growth of skin cancer in B16 mouse melanoma model [149, 150].

In summary, the antitumorigenic effect of CPZ in different cancers frequently involves common molecular/cellular targets that are known to be involved in cancer formation. At the same time, the CPZ’s diverse cellular targets and mechanisms uniquely confer the drug a multiscale antitumorigenic therapeutic potential which has not been fully harnessed yet.

## EFFECT OF CPZ IN ANIMAL CANCER MODELS

Studies in animal models overwhelmingly recapitulate the antitumorigenic effect of CPZ that emerged from *in vitro* studies. One of the earliest studies of the effect of CPZ *in vivo* was conducted by Van Woert and Palmer in 1969 [150]. CPZ was injected intraperitoneally into mice transplanted with B-16 and Harding-Passey mouse melanomas. Examination of the tumors excised 18 days post-transplantation revealed about three-fold decreases in the weight of the tumors treated with CPZ in comparison with the CPZ-untreated controls. Shortly after this study, the anti-tumor effect of CPZ was tested in hamsters by Levij and Polliack in 1970 [152]. Tumor growth was induced by painting (topically applying) different chemical carcinogens to cheek pouches of hamsters in the absence and presence of CPZ. Histological examination of tissue cross-sections 9 and 12 weeks after the treatment indicated that animals painted with both carcinogens and CPZ showed drastically decreased levels of tumorigenesis compared to animals exposed only to carcinogens, including almost complete inhibition of carcinoma formation. These early studies on animal models yielded promising results and prompted many follow-up *in vivo* studies aimed to elucidate CPZ’s anticancer potential and mechanism.

Multiple studies further utilized mice xenograft models to investigate CPZ’s therapeutic potential for different cancer types, including sarcoma [42], colorectal cancer [93], glioma [82], leukemia [147], and oral cancer [45]. These studies showed that CPZ treatment in mice bearing various tumors significantly increased animal survival time [82, 147], quelled tumor growth [45, 93], and caused tumor regression [42]. Importantly, multiple studies reported no significant difference in mice weight between CPZ-treated and control groups, suggesting that CPZ treatment has no adverse effects on body weight [45, 82, 93]. Interestingly, Aas and coworkers found that in rats transplanted with RG2 neuroblastoma cells, CPZ enhanced effect of the anti-neoplastic drug, 1,3-bis(2-chloroethyl-l)-nitrosourea (BCNU), on inhibiting the transplanted tumor growth [151]. In addition, Lee and coworkers found that CPZ enhanced the anticancer effect of pentamidine, an antiparasitic agent, in mice transplanted with A549 lung cancer cells [75]. The synergistic effect of CPZ was also observed in several *in vitro* studies, as discussed above. Therefore, future studies should further explore CPZ’s anticancer potential as a stand-alone medication, as well as in combination with existing therapies.

In addition to the rodent xenograft models, CPZ’s antitumorigenic effect has also been demonstrated in zebrafish. Earlier this year, Jhou and coworkers found that CPZ treatment decreased tumor growth in zebrafish larvae grafted with oral cancer tumors [45]. Notably, CPZ treatment had no observable effect on larval development. Consistent with this report, an earlier study also reported no effect of CPZ on zebrafish development at clinically relevant levels [93]. Taken together, the studies in animal models strongly support antitumorigenic potential of CPZ demonstrated by *in vitro* studies. Furthermore, animal xenograft studies suggest that repurposing CPZ for cancer treatment as a stand-alone medication or in combination with other cancer drugs should be well tolerated and have low adverse effects.

## POPULATION STUDIES OF THE EFFECT OF ANTIPSYCHOTIC MEDICATION ON CANCER RISK

Population studies on the cancer rate in psychiatric patients administered antipsychotics/antidepressants, including CPZ, are somewhat limited and yielded contradictory results. Additionally, most of the studies do not specify which antipsychotic medication was administered to the patients. In a systemic literature review conducted in 2012, Fond and coworkers concluded that overall the patients with schizophrenia may be less likely to develop cancer than the general population [153]. However, individual studies are controversial and frequently inconclusive. Moreover, the reported differences in the cancer rates between psychiatric patients and normal population are frequently cancer type- and gender-specific.

One of the first relevant population studies was conducted in Denmark based on the analysis of 6168 patients with schizophrenia followed from 1957 to 1984 [154]. The study found an overall decrease in the incidence of cancer in the patients, with especially marked decrease in the rate of respiratory system cancers, prostate cancer in male patients and uterine cancer in female patients. Another Danish study of 25,264 users of unspecified antipsychotic drugs monitored between 1989 and 2002, found decreased risk of rectal cancer in both female and male subjects, decreased risk in colon cancer in females and reduced risk of prostate cancer in male subjects [155]. In agreement with the protective effect of antipsychotics on colorectal cancer in Danish population, a study conducted in Taiwan in 2019, based on the analysis of 34,470 gastric cancer patients and 163,430 non-gastric cancer controls, also found a substantial decrease in the rate of gastric cancer in patients prescribed antipsychotic medication, including CPZ [156]. Another comprehensive study based on the analysis of 31,953 cancer cases in United Kingdom (UK), found a statistically significant dose- and time-dependent decrease in the incidences of colorectal cancer and glioma in the users of tricyclic antidepressants [157]. Unlike the above mentioned studies, a study of 26,996 patents with schizophrenia conducted from 1969 and 1991 in Finland found a slight increase in the overall cancer rate in the patients in comparison with the general population, especially marked increase in lung cancer in male patients [158]. However, the study also found a decrease in the incidence of rectal cancer in female patients, and interestingly, a substantial decrease in cancer incidents among non-schizophrenic siblings and parents of the patients. Similar to the other studies, the primary medication administered to the schizophrenic patients was not indicated.

Perhaps the most controversial is the effect of antidepressants/antipsychotics on the rate of breast cancer. A study of 29,641 female schizophrenia patients receiving antidepressants and 59,282 non-users selected based on Taiwan Insurance Claims Data from 1998 to 2008 found that the risk of breast cancer was almost 2-fold higher in the users of antidepressants [159]. This is by far the strongest report of adverse effects of antidepressants and is in agreement with an earlier study in Danish population that found a slight increase in the incidence of breast cancer in female patients [154]. However, several other studies report no dependence in the breast cancer rate and the use of antidepressant/antipsychotic drugs. For instance, a recently conducted study in Denmark on 4,951 antipsychotic users and 47,643 non-users found no clinically important association between the antipsychotic drug use and risk of breast cancer [160]. Two other earlier studies conducted in Denmark and UK, already mentioned in this section, also found that the rate of breast cancer is unaffected by the use of antidepressants [155, 157]. The cohort size for UK study is comparable to the cohort size of the Taiwanese study, yet they reached different conclusions, and the reasons for these differences are not clear. However, it should be noted that the interpretation of population studies on the effect of antipsychotic medications on cancer incidence is very sensitive to the study design and could be affected by a variety of factors, including genetic differences between populations, environmental influence and differences in lifestyle [161]. Therefore, the aforementioned studies and conclusions must be interpreted with caution. Studies on larger populations, based on robust models taking into account the multitude of cofactors and distinctions in the various antipsychotic drugs are necessary to dissect the link between cancer rates and the use of antipsychotics.

## CLINICAL POTENTIAL OF CPZ FOR CANCER TREATMENT

As reviewed above, CPZ has a potent anti-proliferative effect on many cancer types achieved by affecting a variety of molecular targets and cellular pathways. CPZ also has an additional benefit due to the documented sedative effect that can reduce anxiety, insomnia and other symptoms related to the mental state of cancer patients [162]. Therefore, there is a strong rationale for repurposing CPZ for treatment of cancer. Development of new cancer drugs can take many years, and clinical testing can be halted at any stage in the event of unexpected toxicity and safety concerns, making a new drug development a risky and costly endeavor. As an FDA-approved drug that has been used for many decades to treat psychiatric patients, CPZ could be repurposed for the treatment of tumors where the molecular mechanisms of CPZ’s action are well understood, and preclinical and clinical studies support benefits of its administration.

Despite the potential for cancer treatment there are also known side-effects of CPZ that must be considered. CPZ can cause hypertension, bradycardia and ventricular arrhythmias [163]. Therefore, patients have to be monitored for the potential cardiac side-effects. Additionally, CPZ can cause weight gain [164], sexual dysfunction [165], movement disorders, including dystonia and tremors [166], and increase prolactin levels in females, leading to menstrual irregularities [167]. However, many of these symptoms are reversible once the treatment is discontinued [164–166]. The severity of the side-effects depends on the dose and the duration of the treatment. Therefore, fine-tuning CPZ’s dose and duration for optimal treatment of cancer patients, exploring combination therapy with CPZ and other existing therapies and cancer type-specific effects of CPZ should be the focus of future clinical studies. While this review is focused on CPZ, the founding member of the phenothiazines family of drugs with arguably the most studied effects in cancer, many other phenothiazines have been shown to possess anticancer properties as well [43, 168]. Consideration of other phenothiazines that may have a more favorable balance between their antitumorigenic activity and potential side-effects is also a valuable strategy for drug repurposing. Notably, a Phase II clinical trial involving a combination of CPZ and temozolomide for glioblastoma treatment was recently initiated [169]. This and other future clinical studies will shed light on the therapeutic potential of CPZ for cancer treatment.

## CONCLUSIONS

Unequivocally, CPZ is a potent antitumorigenic agent acting through a diverse range of molecular targets and cellular pathways. The rationale for repurposing CPZ for cancer treatment is strong. However, further studies on the molecular and cellular mechanisms of CPZ’s action, and their interdependence are necessary to attain a comprehensive understanding of CPZ’s action in cancer. More refined population studies, specifically focused on the effect of CPZ on cancer patients will also shed light on the therapeutic potential of this drug for cancer therapy. Finally, clinical studies involving dose- and treatment duration-dependence of the CPZ’s effect in cancer are necessary to evaluate a therapeutic potential of repurposing CPZ for cancer treatment.
